# Combined Complementary and Alternative Therapies for the Management of a Breech Fetus: A Feasibility Study

**DOI:** 10.1055/a-2639-7353

**Published:** 2025-07-08

**Authors:** Shilpa Babbar, Karen B. Williams, Lisa Vawter

**Affiliations:** 1 Division of Maternal-Fetal Medicine, Department of Obstetrics, Gynecology and Women's Health, University of Minnesota, Minneapolis, Minnesota; 2 Department of Bioinformatics, University of Missouri Kansas City, Kansas City, Missouri; 3 Lisa Vawter Chiropractic Wellness for Women, Overland Park, Kansas

**Keywords:** yoga, breech, spinning babies, chiropractic care, pregnancy

## Abstract

**Objective:**

Cesarean rates are rising in the United States, especially for breech presentations, which complicates 3 to 4% of term pregnancies and contributes to maternal morbidity. Complementary and alternative therapies (CT) like moxibustion, chiropractic, and hypnosis have been suggested as noninvasive options to encourage fetal version. This study assessed the feasibility and acceptability of combined CT for breech management.

**Study Design:**

Women aged > 18 with a singleton breech fetus at 34 to 37 weeks engaged in three study visits within 2 weeks. The intervention included therapies such as Spinning Babies techniques, yoga postures, mindset techniques, and chiropractic adjustments. Stress levels were assessed pre- and postintervention.

**Results:**

Of 24 referrals, 11 completed the study. No fetuses converted to vertex, but participants reported significantly reduced stress ( *p* = 0.02). After the intervention, 54.5% opted for an external cephalic version, with a 50% success rate leading to two vaginal deliveries. All participants found the program beneficial, reporting high satisfaction with program duration, structure, and exercises.

**Conclusion:**

Although fetal version was not achieved, this CT-based program significantly reduced maternal stress, suggesting its value as an emotional management tool in breech pregnancies. Larger trials are needed to evaluate its efficacy in promoting fetal version and improving maternal outcomes.

**Key Points:**

- Approximately 86.9% of breech fetuses are delivered by cesarean section.
- Complementary therapies are often employed for a breech fetus with varying success.
- Combined complementary therapies for a breech fetus led a significant reduction in stress.

In the United States, there has been a recent rise in women being delivered by cesarean section (C-section) to the current rate of approximately 33%. Although a C-section can be a lifesaving surgery, it comes with an increased risk of maternal morbidity when compared with a vaginal delivery. The top three reasons for performing a C-section are: arrest of labor (34%), nonreassuring fetal heart rate tracing (23%), and malpresentation (17%). 1

Breech presentation, the most common malpresentation, at 37 weeks onward, is estimated to complicate 3 to 4% of pregnancies and approximately 86.9% of persistent breech presentations are delivered by C-section. 1 2 Etiologies for a breech fetus in the third trimester include conditions that change the vertical polarity of the uterus such as a uterine malformation, fibroids, or lack of laxity of the maternal abdominal wall. Conditions that affect the ability of the fetus to turn vertex such as fetal congenital anomalies, abnormal amniotic fluid volumes, or prematurity, also increase the risk. In 2000, a large multicenter randomized trial, the Term Breech Trial, demonstrated significantly lower perinatal and neonatal morbidity and mortality when women underwent a planned C-section for breech compared with a planned vaginal delivery. 2 Hence, the current American College of Obstetricians and Gynecologists recommendation is to perform a C-section as the preferred mode of delivery for a breech fetus. However, an external cephalic version (ECV), as a mean to reduce the rate of C-sections, is standard of care and should be offered to all women who have a breech fetus after 37 weeks who desire a vaginal delivery and have no contraindications. 3 When an ECV is performed successfully, women are more likely to undergo a vaginal delivery, have a lower likelihood of developing complications from delivery, and tend to have a shorter hospital stay. 3

The application of complementary and alternative therapies (CT) in medicine is increasingly common and the literature to support its use in pregnancy is also widely prevalent. Moxibustion, chiropractic techniques, massage, yoga, hypnosis, acupressure, and acupuncture are common CAM therapies used in pregnancy for a variety of reasons, including the management of breech fetuses. 4 5 6 7 8 9 10 11 Traditional Chinese medicine proposes the utilization of moxibustion—the burning of moxa, a root of the herb Artemisia vulgaris, near the BL67 point (outer aspect of pinky toe)—to turn breech fetuses. In 2012, a Cochrane Review 4 that included eight randomized controlled trials (RCTs) found limited evidence to support the use of moxibustion for breech fetuses. When compared with no treatment, moxibustion did not significantly reduce the number of breech fetuses ( *p* = 0.45); however, when combined with acupuncture (at the same BL67 point) or postural techniques, a significant reduction in breech fetuses and C-sections occurred. 4 Since this publication, two more RCTs were published that did not show a significant reduction in breech fetuses with moxibustion. 5 6 Although most of these RCTs recruited patients at 32 to 36 weeks' gestation, the duration of the intervention varied, ranging from twice daily to two times a week, which may explain the discrepancies in conclusions.

In 1978, Dr. Larry Webster developed the “Webster technique,” a chiropractic approach for correction of sacral movement restriction utilizing the chiropractic adjustment and soft tissue work to improve the neurobiomechanical function of the pelvis. 7 When employed, practitioners noticed a “side effect” of the technique was conversion of a breech fetus to a vertex position. In theory, by correcting motion restriction of sacrum or sacroiliac joints and by releasing tension and balancing pelvic muscles and uterine ligaments, this technique facilitates more tissue mobility surrounding the uterus and the pelvis, thus allowing the fetus to move into optimal positioning for labor. 7 This technique is taught by the International Chiropractic Pediatric Association in a comprehensive 13-hour hands-on seminar. Certification requires passing the Webster Proficiency Exam, which includes three components: a practical assessment, a written test, and a principles and practice agreement. Successful completion of all three parts is necessary to achieve Webster Certification. 8 It remains in the scope of practice for a chiropractor as a technique to assess and correct maternal pelvic imbalance and uterine constraints and not as an obstetric procedure to correct the breech fetus. A survey of licensed chiropractors who provide care for pregnant women demonstrated that all the respondents ( *n* = 112) had performed the Webster's technique within the past 6 months with a self-reported success rate of approximately 90%. 9 Currently, there are no RCTs that have assessed the effectiveness of this technique and all assumptions are based on case reports and expert opinion. 7

Relaxation therapies may play a vital role in the management of breech fetuses, as some believe that stress, activation of the sympathetic nervous system, and tension in muscles may prevent a fetus from turning vertex. Hypnosis, a deeply relaxed state, has been assessed in two separate studies. 10 First, a case series of 100 women were offered up to 10 hours of in-person hypnosis after 37 weeks. When compared with 100 matched controls, the spontaneous conversion rate was significantly higher (81 vs. 26% in the control group; *p* -value not provided) and C-section rate was significantly lower in the hypnosis group. 11 Implementing 20 minutes of hypnosis right before an ECV was also shown to significantly increase the success rate of the ECV (41.6 vs. 27.3% of controls; *p* < 0.05). 12

Theoretically, massage and yoga are two CAM modalities that can also lead to relaxation of muscles supporting pregnancy and assist with conversion of a breech fetus. However, there are no peer-reviewed studies assessing their specific effects for this indication. Spinning Babies, a program created by a midwife Gail Tully, promotes the use of specific postures and practices, similar to yoga and the Miles Circuit, 13 during pregnancy to facilitate turning of a breech fetus. 14

The causes of a breech fetus are multifactorial; therefore, a one-size-fits-all approach is not pragmatic. The evidence to support the use of CAM therapies is mixed; thereby, there is not a singular best CAM therapy to implement. Therefore, we devised this study to offer a comprehensive and individualized approach to the breech fetus incorporating several CAM modalities.

We hypothesized that implementation of a combination of complementary and alternative therapies (CCAT) over a short duration of time may result in a decrease rate of breech fetuses after 37 weeks. Our primary objectives include: (1) to determine the feasibility of implementing a CCAT approach to evert a breech fetus and (2) to assess the acceptability of CCAT as measured by patient perception and percentage of patients who opt to enroll in the study. Our secondary objectives include: (1) to obtain preliminary measures of effectiveness to determine if a CCAT for the management of a breech fetus will result in a decreased rate of breech fetuses at term and (2) to determine the rate of C-sections for breech fetuses at term in women who engage in this program.

## Methods

### Trial Design

This is a single-group, feasibility, pilot study.

### Participants

We included women who were ≥18 years of age, English speaking with a singleton, well-dated pregnancy by first trimester ultrasound or known/sure last menstrual period with a fetus in the breech position. These women were also included if they planned to deliver at our hospital to allow for collection of delivery information from our electronic medical records.

Our exclusion criteria included the following: any unstable maternal or fetal condition that requires urgent delivery, any contraindication to vaginal delivery (i.e., placenta previa, placenta accreta, macrosomia, etc.), evidence of labor (i.e., regular contractions, cervical dilatation), women with known uterine malformations, fetal anomalies, or aneuploidy. Those who would not be a candidate for an ECV at the time of their ultrasound in our clinic, i.e., fetal growth restriction, low amniotic fluid volume, premature rupture of membranes, multifetal gestation, nonreactive nonstress test, nuchal cord, etc., were also excluded.

Participants were recruited from the Maternal-Fetal Specialists (MFS) clinic, a high-risk pregnancy consultative practice that relies on the referrals from obstetrics and gynecology physicians who plan to deliver their patients at the main hospital. Participants were referred to our clinic when they were determined to have a breech fetus on ultrasound examination in their physician's office between 34 ^0/7^ and 37 ^6/7^ weeks gestation. Patients are routinely sent to our MFS clinic for evaluation and management of breech fetus including performance of ECVs for breech fetuses when indicated. However, the ECV was not included as an intervention for this research study.

### Intervention

The intervention was conducted over three study visits. Study visit number 1 was initiated on the day that they were referred to our clinic for evaluation. Each participant had a formal ultrasound evaluation using a GE Voluson ultrasound machine (GE Healthcare, Chicago, IL) to determine fetal position, estimated fetal weight, amniotic fluid assessment, placenta location, and to evaluate for any contraindications to an ECV. An intake form was also completed that included questions about their obstetric, nonobstetric, physical activity, and pain history. We also inquired about their personal use of integrative therapies before or during the current pregnancy. A 9-question survey regarding their stress levels over the past 2 weeks was also completed. After obtaining verbal and written consent, the participant met with a licensed chiropractor who specializes in pregnancy to initiate the intervention. Upon reviewing their history and performing a physical assessment, a soft tissue treatment and a chiropractic adjustment were performed if deemed necessary.

The therapies provided during the intervention were divided into three categories: Spinning Babies, Yoga, and mindset techniques with lifestyle modifications. Participants were advised to come with a partner or support person and were taught 3 Spinning Babies techniques, which included belly sifting, side lying release, and forward-leaning inversion. Yoga postures incorporated into our intervention were cat–cow pose, puppy pose, supported bridge pose, child's pose, down dog, and hip figure of 8s. The mindset component of the intervention included visualization, meditation, a list of positive affirmation mantras, and lifestyle modifications that focused on being mindful of posture when sitting, standing, or engaging in activities of daily living. A booklet was created with instructions for all interventions and was provided to each participant ( Supplementary Material S1 ). Acupuncture and moxibustion were excluded from the intervention because the chiropractor was not trained in these modalities at the time. Participants were encouraged to practice the techniques at home daily or as frequently as they felt comfortable between visits. This study visit lasted approximately 2 hours, which included the ultrasound component.

The second study visit took place 3 to 5 days after the first visit. At this visit, a bedside ultrasound using the Butterfly iQ handheld ultrasound probe (Butterfly Network Inc., Guilford, CT) was performed by a Maternal–Fetal Medicine physician (S.B.) to determine fetal position. The participant then met with the chiropractor to review their home practice and engaged in exercises that were previously taught and underwent soft tissue work and Chiropractic adjustment. If the fetus was vertex, new yoga postures were taught to the participant to help keep the fetus in a vertex position. These included standing lunge with or without chair support, malasana (deep squat), goddess pose, and floor lunges. Participants were again encouraged to practice these techniques at home before their next visit.

Study visit three took place 3 to 5 days after study visit number 2. The same intervention occurred as study visit number 2. These appointments lasted approximately 1 hour each. At the end of study visit number 3, a feedback survey was completed by the participants. The survey had seven questions assessing the program and each study visit and contained the same stress survey that was completed prior to the initiation of the intervention. Delivery data were collected by chart review of the electronic medical record.

### Statistical Analysis and Sample Size

Statistical analysis was conducted with SPSS v28.0.1. Descriptive statistics were obtained on the data that were collected. Wilcoxon matched-pairs signed-ranks test was used to compare stress levels before and after the intervention. Our sample size was estimated pragmatically given the feasibility nature of the study. A brief review of our labor and delivery unit statistics between July to December 2021 was performed. During that 6-month timeframe, there were approximately 400 births per month. Of those, C-section for breech occurred at a rate of 5%. Therefore, we anticipated that during the 6-month feasibility study, there would be approximately 120 women with breech fetuses eligible for participation. As our gestational age for recruitment began at 34 weeks instead of 37 weeks, we anticipated that approximately 48 to 50 women referred to our clinic with a breech fetus who would opt to participate in the study.

## Results

This feasibility study was conducted from February 1, 2023 to June 15, 2023. It was initially planned to be a 6-month study period; however, the study period was truncated at 4.5 months of study period due to a lack of institution research support for coverage during maternity leave for one of the primary investigators. As this study was based on referrals from obstetric providers, taking a hiatus to return and complete the 6-month study period was not feasible.

A total of 24 women were referred to our clinic for the management of a breech fetus during the study period. Two women were greater than 38 weeks at the time of their initial visit, which excluded them from our study. Six women declined to schedule an appointment in our clinic, because their fetus was vertex and intervention was not needed. We had a total of 16 women present to our clinic for ultrasound evaluation with the intention of participating in our study. Of these women, 5 were excluded ( *n* = 3 had a vertex fetus, *n* = 2 had nuchal cord), leading to 11 women who enrolled and completed the study intervention. No participants declined to participate in the study after hearing about the details of the study intervention.

The mean maternal age of participation was 29 years. Most of our participants were of white/European race (91%), married (91%), with a college degree (82%) and primiparous (64%); Table 1 ). The majority of women ( *n* = 8, 73%) had engaged in multiple CAM therapies prior to pregnancy as well as during pregnancy, which included acupuncture, acupressure, chiropractic care, massage, yoga, and meditation.

**Table 1:** Baseline demographics

Characteristic	N = 11 N (%) or mean ± SD
Age (years)	29.1 ± 3.7
Race	
White/European descent Black/African descent Asian Mixed Other	10 (91) 0 0 1 (9) 0
Latin/Hispanic ethnicity (yes)	0
Marital status	
Never married Married/Living with partner Divorced/widowed/separated	1 (9) 10 (91) 0
Education level	
Less than high school High school graduate Some college College graduate Missing data	0 0 1 (9) 9 (81.8) 1 (9)
Primiparous (n %)	7 (63.6)
Previous C-section	1 (9)

The average gestational age at visit number 1 was 36.8 weeks. All participants completed this visit and were advised to complete home exercises from all three intervention categories. The average gestational age at visit number 2 was 37.3 weeks. All participants completed this visit and all fetuses were found to be breech at this visit. Eight (72%) women reported engaging in daily home practice of the recommended therapies, whereas 27% ( *n* = 3) stated that they engaged in therapies at home but not daily. Ten of the 11 participants were advised to complete home exercises from all three intervention categories at the end of visit number 2. The average gestational age at visit number 3 was 37.8 weeks. The completion rate for visit number 3 was 73% (8/11). All fetuses remained breech at this visit.

After completing the intervention visits, a significant reduction in stress levels pertaining to pregnancy and breech positioning was found. Specifically, reduced stress related to having a breech fetus ( *p* = 0.02), having a C-section for a breech fetus ( *p* = 0.01), the process of labor and delivery ( *p* = 0.04), and recovery after delivery ( *p* = 0.01) occurred. There were no significant differences in other stress factors such as home life, work life, family, finances, or general health ( Table 2 ). Half of the participants chose to undergo an ECV ( *n* = 6; 54.5%) at the completion of the pilot study. The average gestational age at ECV was 38.3 weeks. The ECV procedure had a 50% success rate ( *n* = 3), and all three fetuses remained vertex at the time of induction of labor. Two (18%) of these women delivered vaginally and the other participant underwent a C-section for failure to progress. The remainder of the participants underwent a C-section at delivery (82%). The average gestational age at delivery was 39.4 weeks for the cohort with an average neonatal birth weight of 3644.5 g. The 5-minute Apgar score was 9 for all participants, and there were no neonatal intensive care unit admission or neonatal deaths.

**Table 2:** Pre-post intervention stress survey responses

Stressor	Preintervention Median (SIQ)	Postintervention Median (SIQ)	*p* -value
Having a breech fetus	3 (2)	1.5 (2)	0.02
Having a C-section if my fetus is breech	3 (2)	2 (1)	0.01
My life at home	1 (1)	0 (0)	0.10
My general health during this pregnancy	1 (1)	0.5 (1)	0.73
My life at work	1 (2)	0.5 (2)	0.71
My family	0 (1)	0 (0)	0.32
The process of labor and delivery	1 (2)	1 (0)	0.04
My current financial situation	1 (1)	0 (1)	0.10
My recovery after delivery	3 (1)	1 (1)	0.01

0= no stress, 1 = slight stress, 2 = somewhat stress, 3= mod stress, 4= extreme stress.

Wilcoxon Matched-Pairs Signed-Ranks Test used for analysis.

Table 3 provides the feedback survey responses. Although the fetuses remained breech at the end of the intervention, all women felt the intervention was beneficial. The majority felt that the overall study program duration was sufficient (73%), as was each study visit. Over 90% of participants reported that the homework was sufficient with one participant feeling like it was too much work to do. We received overwhelming positive open feedback regarding the program. Several women wished they had started the intervention earlier.

**Table 3:** Feedback survey

Questions	Responses N (%)
1. How beneficial do you think this program was for you?	
□ Waste of time/completely useless □ Somewhat beneficial □ Very beneficial □ Excellent- best new thing!	0 0 3 (27) 8 (73)
2. What did you think about our study program length? (i.e., total of 3 study visits)	
□ Not enough time □ Perfect length of time □ Too many visits □ Other (please specify): i. Did not get to finish program (due to delivery) ii. Wanted 4 visits	1 (9) 8 (73) 0 2 (18) 1 1
3. What did you think about the body balancing tools homework?	
□ Not enough for me to do at home □ Just enough for me to do at home □ Too much work to do at home □ Other (please specify):	0 10 (91) 1 (9) 0
4. What did you think about the duration of your first study visit?	
□ Not enough time □ Perfect length of time □ Too long of a visit □ Other (please specify):	0 11 (100) 0 0
5. What did you think about the duration of your 2 ^nd^ study visit?	
□ Not enough time □ Perfect length of time □ Too long of a visit □ Other (please specify): i. Didn't get to finish the program * □ Not applicable i. I didn't need the appointment because … ii. I missed the appointment because …	0 10 (91) 1 (9) 1 0
6. What did you think about the duration of your 3 ^rd^ study visit?	
□ Not enough time □ Perfect length of time □ Too long of a visit □ Other (please specify):__________________________________________________ □ Not applicable	0 7 (64) 0 4 (36)
i. I didn't need the appointment because: 1. I delivered 2. I scheduled an ECV instead of the 3 ^rd^ visit ii. I missed the appointment because…	2 (50) 2 (50)
7. Open ended feedback responses:	
Think this is a great idea, no recommendations for improvement	
Starting off earlier	
Liked the flexible appts	
Loved the program	
Great program, felt supported, gave exercise and lifestyle skills, offer to any mom with breech baby	
Great program, love holistic parts of it	
Maybe have a min & max time per day	
Wished she would have known baby was breech sooner so could have tried intervention sooner	
Offer earlier	
100% think this should be integrated w/services	

* This participant was delivered after completing her 2 ^nd^ visit and did not complete the entire program.

## Discussion

In this feasibility pilot study, we assessed the implementation of a comprehensive complementary and alternative therapies intervention for managing breech fetuses between 34 and 37 weeks of gestation. Although none of the fetuses turned to a vertex position by the end of the intervention, participants reported a significant reduction in stress levels associated with concerns about their breech fetus, the likelihood of a C-section, and the overall labor and recovery process. These findings suggest that CCAT may have a role in reducing maternal anxiety, even if it does not directly influence fetal positioning. The positive responses to the program, 100% enrollment rate for eligible participants, and high completion rate of visits demonstrated acceptability and feasibly of implementing this approach.

The findings of our study provide an interesting contrast to the existing literature, which suggests that complementary therapies, such as acupuncture, moxibustion, and chiropractic techniques, may facilitate fetal turning in some instances, although the evidence remains mixed and dependent on specific conditions (e.g., gestational age, fetal size, and maternal anatomy) (Cochrane Review, 2012). Notably, the success rates of chiropractic methods, like the Webster technique, have primarily been based on case reports and expert opinions, with success rates up to 90% reported by practitioners. 9 However, our study did not observe such high rates of spontaneous vertex conversion, indicating that further RCTs with larger sample sizes are needed to validate these anecdotal success rates.

One of the key findings of this study was the significant reduction in stress levels among participants postintervention. This outcome is noteworthy, as elevated maternal stress is associated with adverse pregnancy outcomes, including preterm labor and increased C-section rates. By introducing relaxation-focused modalities such as yoga, meditation, and postural techniques (e.g., Spinning Babies), the intervention may have indirectly influenced participants' mental health and preparedness for delivery. This is in alignment with existing literature indicating that mind–body techniques, including hypnosis and yoga, may enhance maternal well-being during pregnancy. 10 15

An ECV, the application of external pressure to the maternal abdomen to rotate a fetus in an either forward or backward roll into a vertex position, is currently the standard of care, but widely underused as women commonly decline to proceed with an ECV after adequate counseling. 1 Although there are no absolute contraindications to an ECV, there are factors that decrease the likelihood of success, including low amniotic fluid volume, fetal growth restriction, less than 37 weeks' gestation, obesity, anterior placenta, advanced cervical dilatation or evidence of labor, low fetal station, and nulliparity. 3 The success rate of an ECV varies widely between the range of 16 to 100% with an average of 58%. Our study demonstrated a 50% success rate of ECV, of which 66% delivered vaginally. This is consistent with data obtained from our institution's labor and delivery unit from July to December 2021 showed a 50% success rate for ECVs.

## Strengths and Limitations

The strength of our feasibility study includes implementing a multiprong approach to everting a breech fetus. This holistic approach allows for an individualized care plan tailored to each participant's specific needs, preferences, and physical conditions. This also allows for addressing multiple factors, which may be influencing fetal position, as well as improved acceptance and engagement by the participants. The therapies are noninvasive with minimal to no risk to the pregnancy and are easy to learn. Learning self-driven therapies, such as mindfulness, meditation, and lifestyle modifications, can be highly beneficial as it empowers mothers with the skills they can use whenever needed, both during the pregnancy and postpartum period and in parenthood. In addition, the intervention was delivered by a single provider well-versed in all the implemented therapies. This streamlined approach enhanced the patient experience by eliminating the need for multiple practitioners, ensuring continuity of care and a comprehensive understanding of the patient's history, body, and treatment plan.

This feasibility study also has several limitations. The sample size was small ( *n* = 11), and the study duration was truncated to 4.5 months due to institutional constraints, resulting in fewer referrals than anticipated. As the study relied on external referrals to our clinic, we addressed this at the onset of the study by meeting with each referring group to discuss the study details and flyers were provided for display in their clinics; however, we were unable to place a dedicated recruiter in each clinic, making us dependent on individual providers' interest in referring patients. This limited our ability to reach the anticipated target enrollment of 48 to 50 participants, reducing the statistical power needed to detect a significant effect on fetal position change.

The average gestational age at the first intervention visit was 36.8 weeks. Five participants self-reported that their fetus was identified as breech at their second-trimester ultrasound. Given the positive feedback from participants who wished they had started the intervention earlier, future studies could explore the optimal timing and duration of CCAT, including whether initiating the intervention before 34 weeks leads to different outcomes. Furthermore, participants were predominantly of White/European descent, married, and well-educated, limiting the generalizability of our findings to more diverse populations. Future studies should aim to include a larger, more diverse sample and employ an RCT design to provide more robust evidence for the efficacy of CCAT.

## Implications for Future Research and Practice

Despite our limitations, our study provides valuable preliminary data suggesting that a comprehensive CCAT approach is feasible and acceptable for patients with a breech fetus. The significant reduction in maternal stress levels points to the potential mental health benefits of incorporating complementary therapies into standard prenatal care, even if the physical outcomes (fetal position change) are not directly affected.

The varying outcomes across studies, including our own, point to several areas for future investigation. First, it is important to identify which specific factors contribute to the success or failure of CT in facilitating fetal version. Future studies could explore the timing, frequency, and combination of therapies to optimize effectiveness. In addition, exploration of the integration of CCAT with conventional interventions like ECV can be pursued to assess whether these therapies can enhance the success rates of such procedures or improve overall maternal and neonatal outcomes.

## Conclusion

In conclusion, while the CCAT did not result in a change of fetal position in our small cohort, it did significantly reduce maternal stress related to breech presentation and delivery. This suggests that CCAT may serve as a beneficial adjunctive therapy in managing the emotional aspects of a breech pregnancy, highlighting the need for larger, controlled studies to further explore its potential benefits and efficacy in facilitating fetal version.
