# Supplementary material for: Combined Complementary and Alternative Therapies for the Management of a Breech Fetus: A Feasibility Study
Source: AJP Rep. 2025 Jul 8;15(3):e98–e105. doi: 10.1055/a-2639-7353 (PMC12237535; doi:10.1055/a-2639-7353)
Supplement: Supplementary file 1 — Supplementary Material [file 10-1055-a-2639-7353_26506226.pdf]

BODY BALANCING TOOLS

DAILY HOMEWORK	SUN	MON	TUES	WED	THURS	FRI	SAT
**Spinning Babies™ Techniques**							
Belly Lifting							
Side-lying Hip Compression							
Couch Inversion x 3							
**Yoga postures**							
Cat-cow pose							
Puppy pose							
Bridge pose							
Child's pose							
Down dog							
Hip figure 8's							
**Mindset**							
Visualization							
Meditation							
Mantras							
Lifestyle modifications							

Belly Lifting

Do 1-3 times daily for 3-5 breaths

How to do this technique:

1. The support person spreads the fabric across your belly evenly, making sure it's not bunched or pinching anywhere.
2. Move into position on the floor facing a comfortable surface like a chair, couch, or ottoman (hands and knees is fine if nothing is available). Drape your arms over the chair or couch. Relax the upper body and keep the spine in neutral. Settle in.
3. Support person position is stacked above your hips with a solid grip on the fabric. They should bend their knees and brace their core to lift safely.

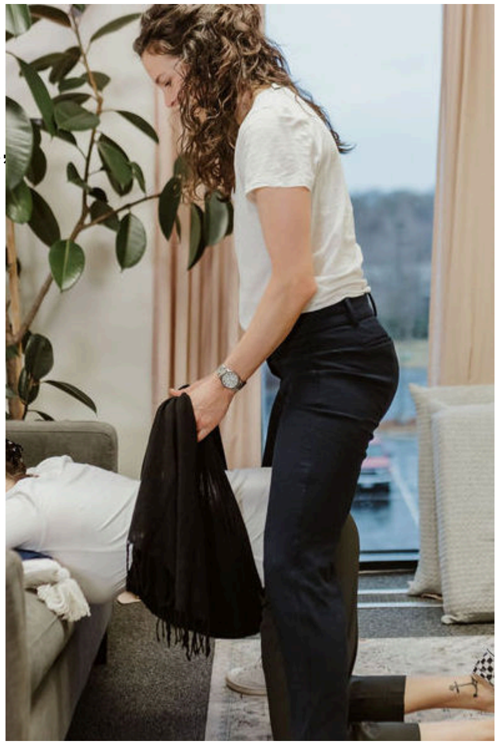

4. Gripping the fabric to pull it taut, partner gently lifts the weight of your belly, checking in for comfort, allowing you to let go and relax fully.
5. Beginning slowly, the partner moves their arms In small circular motions, like wheels on a train or peddling a bicycle. Then gradually increase the speed of the movement if desired.
6. Let go of your low belly, hip and pelvic floor muscles.
7. Communicate with your support person if you want them to go faster/slower, lift more/less. This is a great place to practice working together and exploring what feels good. Make sure each person is regulated and in a place to give/receive feedback, so it is truly relaxing and enjoyable.
8. When it's time to stop, make sure the support person gives you time to prepare and counts down with 3-2-1 as they lowering you down and release the weight of the belly.

Side-lying Hip Compression

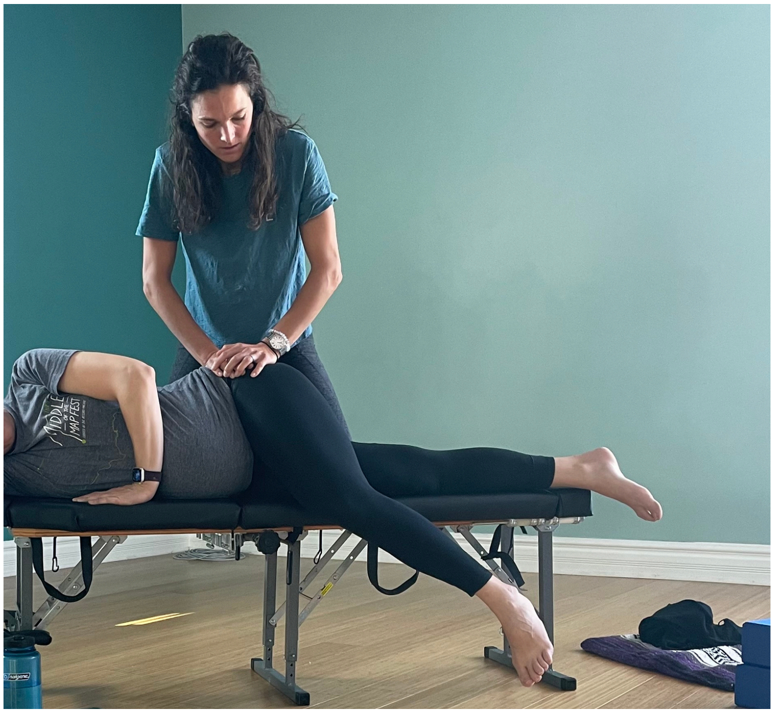

Do both sides daily for 3-5 minutes on each side

How to do this technique:

1. Start in side lying with your hips about 6 inches from the edge of a couch or bed, ensuring your spine is in neutral from your pelvis to your neck.
2. For alignment - stack your pelvis and shoulders, so your aren't twisted or tilted.
3. The support person stands in front (or behind if necessary*) and gently pins your hips to keep you from tipping off.
4. The support person's hand placement is the heel of one palm against the hip bone and the other at the topmost part of your hip (trochanter). Partners should keep a relaxed hand to avoid curling their fingers or gripping into the muscle to hold on. The hands splay toward each other to overlap and provide a broad contact that allows you to fully relax into the position.

6. Take care to check in for comfort with hand placement. Give feedback if adjustments need to made before pressure is applied.

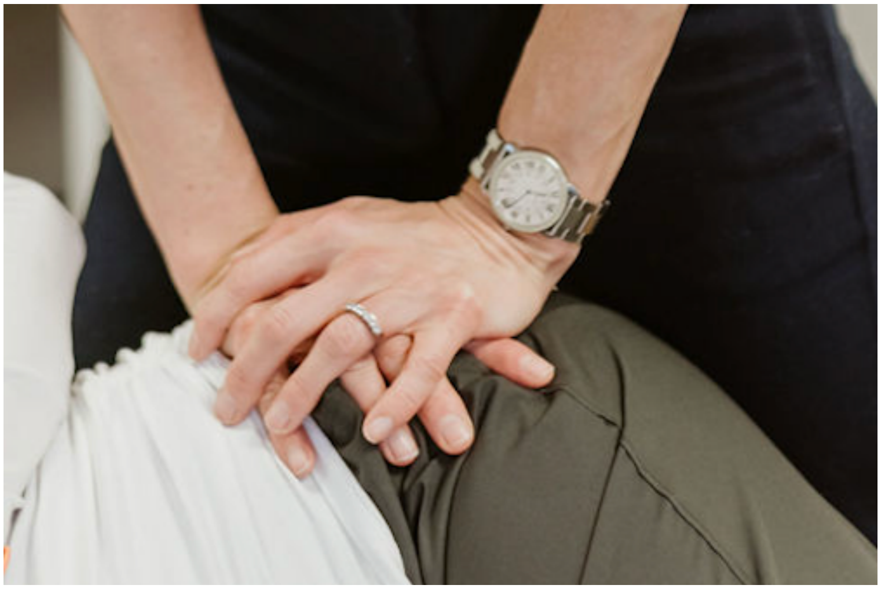

7. Partner begins to add pressure by leaning their body weight over their hands, elbows bent. The pressure should feel secure enough for you to fully relax your jaw, shoulders, low belly, hips, pelvic floor and legs.
8. Ensure the bottom leg is straight, flex the toes toward your nose the whole time.
9. When you are set up, slowly move your top leg forward and dropping your thigh, calf and ankle over the edge. Drape the whole leg to hang off the edge.
10. Recheck your position and make any adjustments needed:
11. Are the hips and shoulders stacked?
12. Is the lower leg straight with toes flexed?
13. Is the leg hanging freely? If the leg rests on the front of the couch or bed, reposition and try again.
14. Switch sides
15. After you finish, stand up and move around, walking will help integrate the balance from your pelvis to your legs.

Couch Inversion

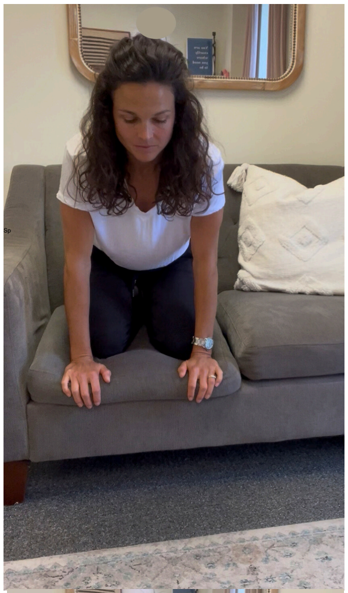

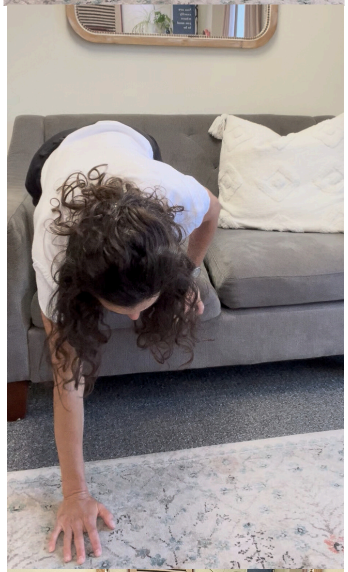

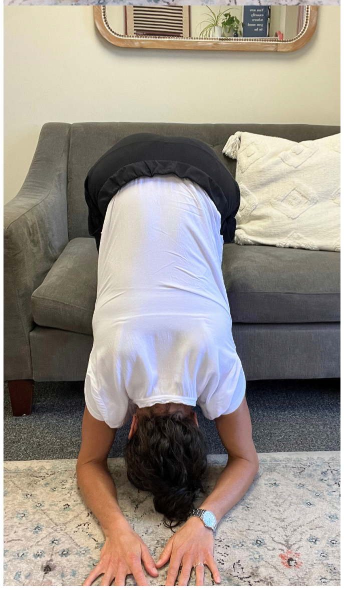

Do1-3 times for 3-5 breaths daily

Off the couch inversion releases tension in the ligaments that attach to the pelvis, womb and cervix and optimizes the space for baby to move and turn.

How to do this technique:
1. The start and end position are the same. Start on your knees with your spine in neutral - on the edge of a sturdy surface like a couch, chair, bed.
2. Securely lean forward and put both hands on the edge of the couch, in a hands and knees position.
3. Slow and controlled lower one hand at a time to the floor. Then, if a steeper inversion is desired, lower down onto your forearms one at a time. Stay close to the couch to stack pelvis over ribs.
4. Press into the floor to activate your shoulders, relax your jaw and tuck your chin to your chest, letting your head hang - nod yes/no to release tension. Do not rest your head on the floor.
5. Let go of your low belly and if able, do a pelvic tilt to release tension in your pelvis and hips.
6.Take 1-5 breaths. Keep your belly loose and shoulders strong. Do not stay upside down too long.
7. Pushing up one hand and then arm at a time, come back up with hands at the edge of the couch. Take a steadying breath. Then come all the way up to kneeling with a straight spine. If you feel overly light-headed here, decrease the time spent inverted.

8. Stay kneeling for a few more breaths. Let the balance you created settle in, this is where the magic happens. Sit back to your heels.
9. Keeping your legs and knees pressed together, swivel your feet to the floor (like a mermaid) to protect your pubic symphysis, maintain balance and pelvic stability.
10. Staying on your hands or adding yoga blocks is a great way to modify the inversion to your comfort. Remember, the goal is relaxation.

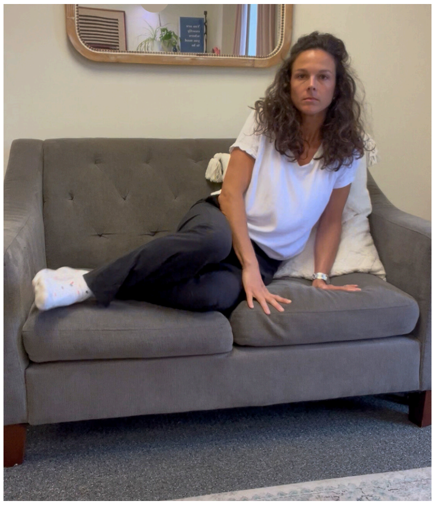

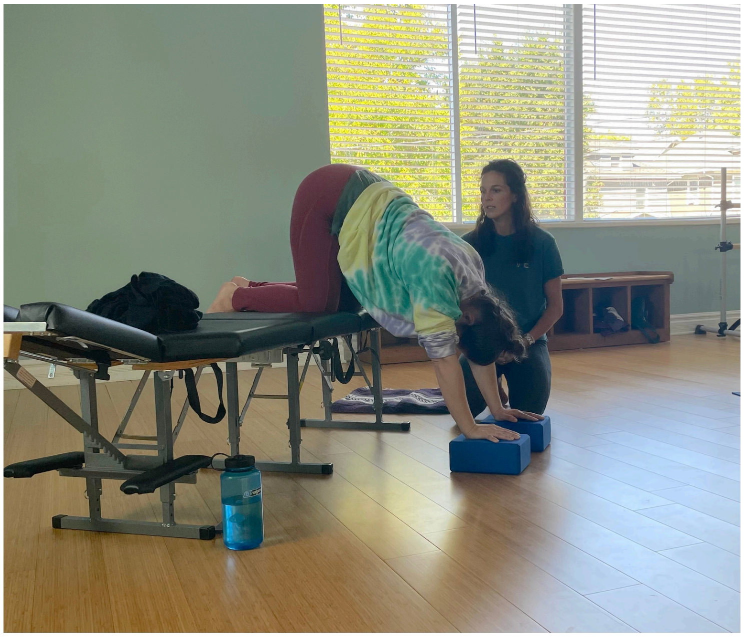

Please visit www.spinningbabies.com for the most up to date information on these practices

Yoga Poses for Breech Presentation

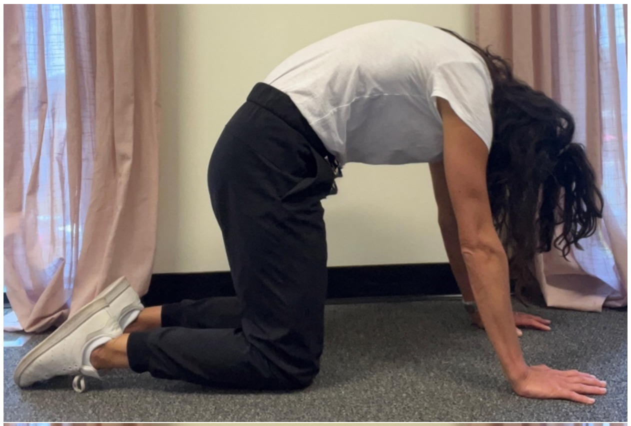

1.Cat Pose
On hands and knees, arch your back to the ceiling and tuck your chin, hold pose for one breath in and out.

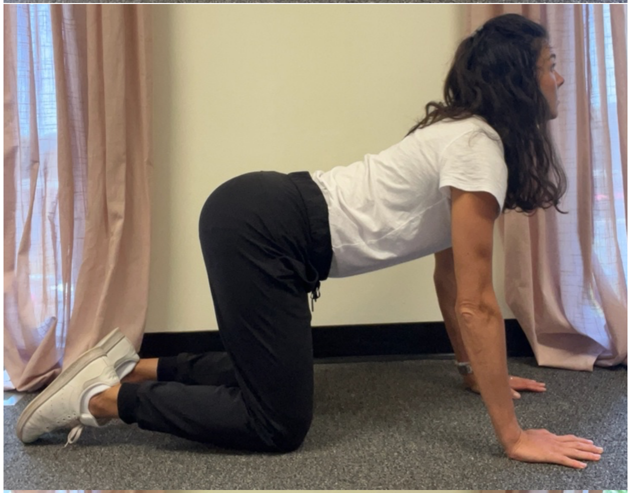

2.Cow Pose
Then gently drop your belly to the floor and look up for one breath in and out.

Repeat 1 & 2 slowly 5 times.

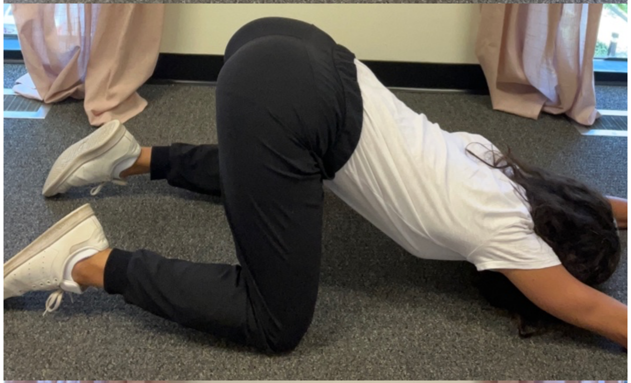

3. Puppy Pose
From hands and knees, walk your hands out and slowly lower your chest and forehead to the ground, keep your booty in the air. Breathe into your belly for 5 deep breaths.

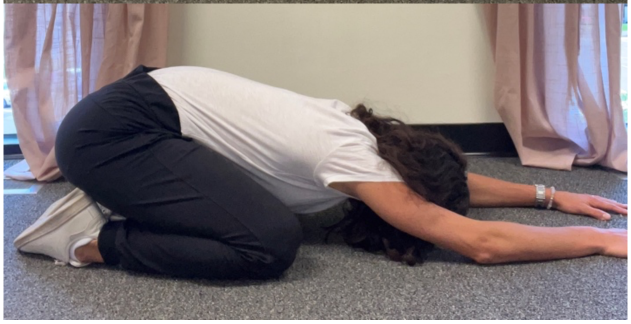

4. Child's Pose
From puppy pose, widen your knees and slowly lower your buttocks onto your heels, keeping your arms stretched out above your head. Breathe into your belly for 5 breaths.

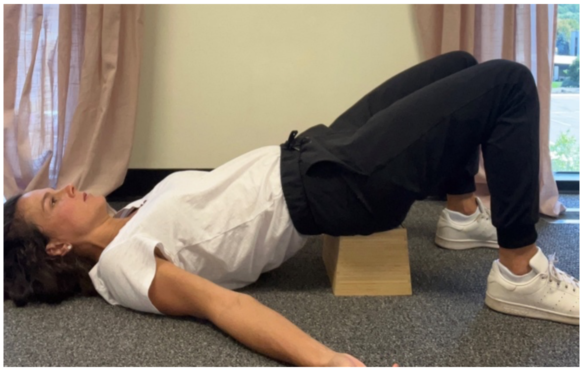

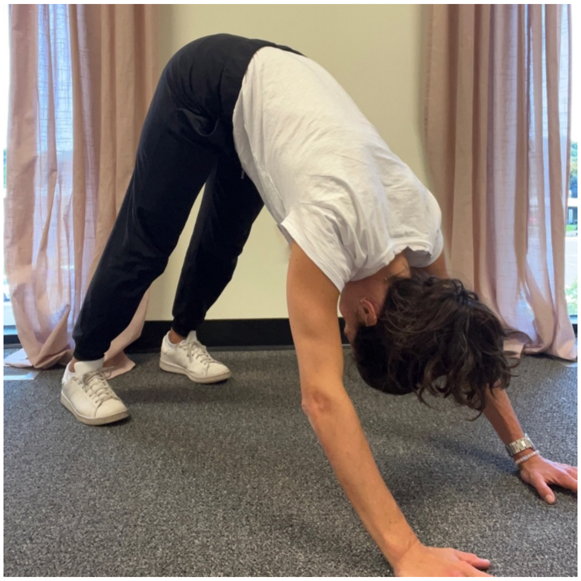

5. Supported Bridge Pose
Lay on your back, knees bent to 90 degrees with feet hip distance apart. Press into your heels and elevate your hips 4-6in from the floor. Slide an object under your sacrum and relax onto it. Take 5 breaths.
To exit the position. Press into heels and lift hips up, slide object out and slowly lower to floor.

6. Downward Dog
From hands and knees, shift weight into shoulders, activate arms and lift your hips to the ceiling. The goal is not straight legs, it is to raise your pelvis above your shoulders. Take 3-5 breaths

VISUALIZATION

Visualization – powerful technique used to “see” the outcome you desire

One way to practice is the following:

- Close your eyes and visualize the space the baby lives—
  - What color is the womb?
  - What does it feel like?
  - How is the baby positioned are they smiling?

- Now visualize the path the baby will take to move head down
  - Moving from head up under your ribs
  - Down along the edge of your uterus
  - Past your hip bone
  - And settling into the space above your pubic bone

MEDITATION

Meditation – a practice to train attention and awareness

Several ways to practice:

- Find a short, guided relaxation meditation on an app
  - I.e. Expectful, Calm, Insight Timer, Headspace

- Box breathing: inhale for 2, hold for 2, exhale for 2, hold for 2
  - Slowly lengthen the duration over time

- Simply inhale for 3 and exhale for 6 (3-5 minutes in duration)

Mantras

✷ My baby and I are safe ✷

☼ I am relaxed and calm ☼

❖ I am already an amazing mom ❖

✦ My baby is the perfect size for my body ✦

✹ I am grateful to my body ✹

⟐ I have wisdom within me to help me respond to the needs of my baby ⟐

★ My body and my baby work together as a team ★

✷ My baby has space to grow and move ✷

☼ I trust my maternal instincts, I know what to do ☼

❖ My body has nourished and protected my baby ❖

✦ I trust my body and my baby ✦

✹ My body is capable and strong ✹

⟐ I encourage my baby to move around in my womb ⟐

★ My body and my baby know what's best ★

✷ I release all tension, I relax all of my muscles ✷

☼ It is possible for my baby to move if they wish to do so ☼

❖ My body is relaxing from my forehead, my shoulders, my belly, my legs and feet ❖

✦ When I relax my body opens and releases tension ✦

✹ I use gravity in my favor to position my baby for an easier birth ✹

⟐ My muscles and ligaments stretch to accommodate my baby ⟐

★ My baby has time to grow and move ★

✷ My baby will be guided by my thoughts ✷

☼ My baby's head will be guided by gravity ☼

❖ I trust my baby to know how to be born ❖

✦ I trust my body to know how to birth my baby ✦

✹ I trust my inner strength to guide me ✹

⟐ I trust myself to remain calm ⟐

★ I am confident ★

✷ I am strong ✷

☼ My mothering instinct is my guide ☼

❖ Everything is going to be ok ❖

✦ I can do hard things ✦

✹ I am proud of my body ✹

⟐ I am my baby's safe haven ⟐

★ My body is amazing ★

Lifestyle Modification Recommendations

**THINK* about your body*

Be mindful of your posture when

a) **Sitting in a chair**
   - ☐ Sit up on your sits bones
   - ☐ Knees below hips (desk, couch, mealtime, car, etc)
   - ☐ Sit symmetrically on both sits bones
   - ☐ Cross legs at ankles or not at all
   - ☐ Upright torso - lift rib cage up off of belly

b) **Standing**
   - ☐ Relax your jaw
   - ☐ Rib cage stacked on top of pelvis
   - ☐ Stand balanced on both legs (pay attention to lateral hip shift)
   - ☐ Unclench glutes
   - ☐ Pelvis in neutral
   - ☐ Slight bend in knees
   - ☐ Relax belly

Notice how you perform Activities of Daily Living (ADLs)

- ☐ How does your body feel when you do certain tasks?
- ☐ How often do you engage in repetitive movements? (ex. always rotating or moving the same direction, shifting weight onto one leg, leaning on one elbow, hunching forward at the computer, etc)

Engage in mindful movement

- ☐ Increase physical activity to at least 20min/day - walk, swim, yoga, etc
- ☐ Hip hinge to pick up items
- ☐ When sitting on the floor, sit in a shin box or 90-90 position
- ☐ Take a movement break each hour with prolonged sitting
  - o (ex. sit for 50 min and stand for 10 min)
- ☐ Shift breath into belly as much as possible
